# A Novel Dipeptidyl Peptidase IV Inhibitory Tea Peptide Improves Pancreatic β-Cell Function and Reduces α-Cell Proliferation in Streptozotocin-Induced Diabetic Mice

**DOI:** 10.3390/ijms20020322

**Published:** 2019-01-14

**Authors:** Yating Lu, Peng Lu, Yu Wang, Xiaodong Fang, Jianming Wu, Xiaochang Wang

**Affiliations:** 1 Tea Research Institute, College of Agriculture and Biotechnology, Zhejiang University, 866 Yuhangtang Road, Hangzhou 310058, China; ytlu@zju.edu.cn (Y.L.); 11616051@zju.edu.cn (Y.W.); 2 Department of Applied Biological Chemistry, Graduate School of Agricultural and Life Sciences, The University of Tokyo, 1-1-1 Yayoi, Bunkyo-ku, Tokyo 113-8657, Japan; zjhzlupeng@hotmail.com; 3 Guangzhou Chinese Medicine Science Technology Co., Ltd., Guangzhou 510000, China; xdfang36@vip.sina.com; 4 Huzhou Jiasheng Tea Co., Ltd., Huzhou 313000, China; jianmingwu2008@sina.com

**Keywords:** dipeptidyl peptidase IV inhibitor, GLP-1, type 2 diabetes, tea, peptide, streptozotocin

## Abstract

Dipeptidyl peptidase IV (DPP-IV) inhibitors occupy a growing place in the drugs used for the management of type 2 diabetes. Recently, food components, including food-derived bioactive peptides, have been suggested as sources of DPP-IV inhibitors without side effects. Chinese black tea is a traditional health beverage, and it was used for finding DPP-IV inhibitory peptides in this study. The ultra-filtrated fractions isolated from the aqueous extracts of black tea revealed DPP-IV inhibitory activity in vitro. Four peptides under 1 kDa were identified by SDS-PAGE and LC-MS/MS (Liquid Chromatography-Mass Spectrometry-Mass Spectrometry) from the ultra-filtrate. The peptide II (sequence: AGFAGDDAPR), with a molecular mass of 976 Da, showed the greatest DPP-IV inhibitory activity (in vitro) among the four peptides. After administration of peptide II (400 mg/day) for 57 days to streptozotocin (STZ)-induced hyperglycemic mice, the concentration of glucagon-like peptide-1 (GLP-1) in the blood increased from 9.85 ± 1.96 pmol/L to 19.22 ± 6.79 pmol/L, and the insulin level was increased 4.3-fold compared to that in STZ control mice. Immunohistochemistry revealed the improved function of pancreatic beta-cells and suppressed proliferation of pancreatic alpha-cells. This study provides new insight into the use of black tea as a potential resource of food-derived DPP-IV inhibitory peptides for the management of type 2 diabetes.

## 1. Introduction

Dipeptidyl peptidase IV (DPP-IV, E.C. 3.4.14.5) is a multifunctional transmembrane glycoprotein that selectively cleaves dipeptides after proline or alanine residues and it is widely distributed in human organs and tissues [1,2,3]. DPP-IV is renowned for its inactivation of two major incretin hormones involved in glucose homeostasis, namely, glucagon-like peptide-1 (GLP-1) and glucose-dependent insulinotropic polypeptide (GIP) [4,5], which stimulate insulin secretion but inhibit glucagon secretion [6]. Type 2 diabetes patients show an impaired response of GLP-1 to oral glucose, and have reduced GLP-1 baseline concentrations versus obese patients without diabetes [7]. In addition, the decreased response of meal-related GLP-1 compared with that of GIP may contribute to the decreased incretin effect in type 2 diabetes [8].

DPP-IV inhibitors are currently being used as a novel therapy in the management of type 2 diabetes [9,10]. Acting as glucose-lowering agents, the primary pharmacological action of the DPP-IV inhibitors is to prolong the active time of the two major incretin hormones (GLP-1 and GIP). Additionally, many other actions of DPP-IV inhibitors have also been reported, including the stimulation of beta-cell survival and islet neogenesis [11], the improvement of beta-cell function [12], the decrease of alpha-cell proliferation [13], and the reduction of total cholesterol [14]. DPP-IV inhibitory chemosynthetic drugs, including sitagliptin, vildagliptin, saxagliptin, etc., have been approved by the Food and Drug Administration (FDA) in the United States since 2006 [9,15,16]. Recently, increased effort has been devoted to finding new natural antihyperglycemic agents, because there are no side effects [17,18].

Bioactive peptides are specific protein fragments that have a positive impact on body functions or conditions, and may ultimately influence health [17]. DPP-IV inhibitory peptides derived from food proteins have attracted particular attention for their ability to prevent hyperglycemia. Hydrolysates approximately 1 kDa in size have been isolated from various natural sources, such as milk proteins, rice bran, amaranth proteins, ham, soybean, and fish proteins, and these hydrolysates display in vitro DPP-IV inhibitory activity [19,20]. Several animal studies have reported the in vivo antidiabetic effects of peptides, but the mechanisms have not been completely elucidated. It is meaningful to enlarge the bioactive peptide database and further explain the mechanisms of these proteins through detailed animal studies.

Tea is a traditional health beverage in China, and its antioxidant, antidiabetic, anti-hypertensive functions have been confirmed. For instance, tea polysaccharides can inhibit alpha-glucosidase, which is related to type 1 diabetes [21,22], and tea polyphenol is one of the effective antidiabetic components in decreasing glucose levels and preventing complications [23,24,25,26]. It is worth noting that the fact that flavonoids exhibit DPP-IV inhibitory activity has been proven [27,28], and of course, tea polyphenol has also been taken into consideration in our pilot study (see Figure S1 online). Nevertheless, there are six types of tea in the Chinese market: green tea, white tea, yellow tea, oolong tea, black tea, and dark tea. Black tea is a fully fermented tea produced and consumed worldwide [29]. Compared with the other types of tea, black tea presented the lowest antioxidant activity, due to the oxidation of flavonoids during fermentation [30]. Because of the protein hydrolysis by endogenous protease during the production process [31], it is highly likely that black tea contains rich bioactive peptides. Considering the previously reported significant inhibitory activity of small peptides, the existence of similar peptides rather than flavonoids in black tea is of great interest in this study.

The present study primarily focused on the identification of DPP-IV inhibitory peptides in black tea and examined their antidiabetic effects on streptozotocin (STZ)-induced diabetic mice. These results should provide insights into the utilization of black tea as a potential source of bioactive peptides for the treatment of type 2 diabetes. Moreover, the influence of DPP-IV inhibitory peptides on islet function and morphology were also further investigated.

## 2. Results

### 2.1. Peptide Identification

The extracts were passed through various membranes with molecular mass cutoffs of 50, 10, 5 and 1 kDa, respectively, and flow-through was collected to detect the inhibition capability. The multiple comparisons demonstrated in Table 1 suggest that the concentration of total tea phenols decreased after using smaller membranes. The concentration of total phenols in the filtrate under 50 kDa was 49.2 ± 0.83 μg/mL, decreasing to 30.7 ± 0.82 μg/mL for total phenols under 1 kDa. No DPP-IV inhibitory activity could be detected when the concentration of tea polyphenols was under 50 μg/mL (Figure S1). However, no significant difference was found in DPP-IV inhibition between different filtrates (>70%). Thus, we proposed that something else like peptides might play a part, in addition to tea polyphenols.

**Figure 1:**
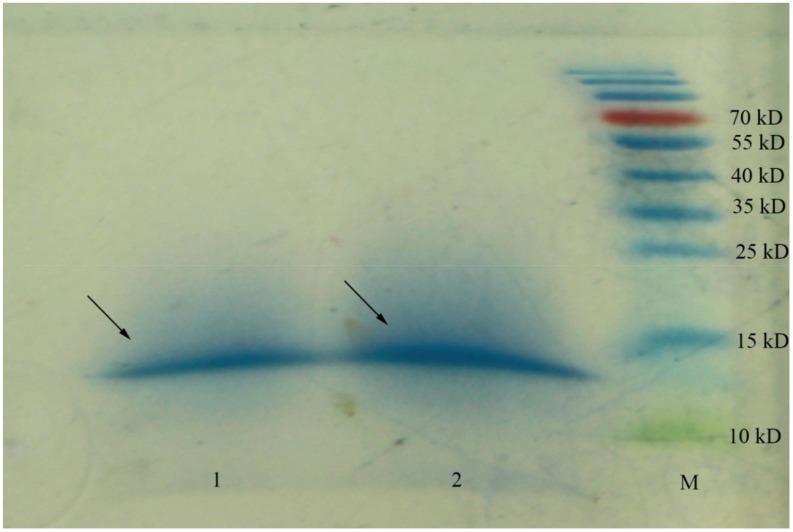
SDS-PAGE gel of Lapsang souchon (LS) extracts obtained from 1kDa membrane. Peptides were revealed using the colloidal Coomassie blue stain. The experiment performed in duplicate was numbered 1, 2. The bands labeled with arrows were used to perform LC-MS/MS analysis. M: molecular mass marker.

To find bioactive peptides around 1 kDa, SDS-PAGE was used to identify and prepare the peptides for LC-MS/MS analysis. The peptides remained aggregated according to Figure 1, as SDS-PAGE could not separate peptides of such small sizes. The unresolved peptides were collected, and four sequences with high ion scores were determined after LC-MS/MS (shown in Table 2; their MS/MS chromatograms were shown in Figure S2). These peptides were 6–13 amino acid residues in length; among them, peptide I and peptide II both contained ten amino acid residues with sequences QTDEYGNPPR and AGFAGDDAPR, respectively. The sequence IDESLR had the lowest theoretical molecule weight at 731.80 Da, and peptide IV (IQDKEGIPPDQQR) is the longest sequence.

### 2.2. Dipeptidyl Peptidase IV Inhibitory Assay

**Figure 2:**
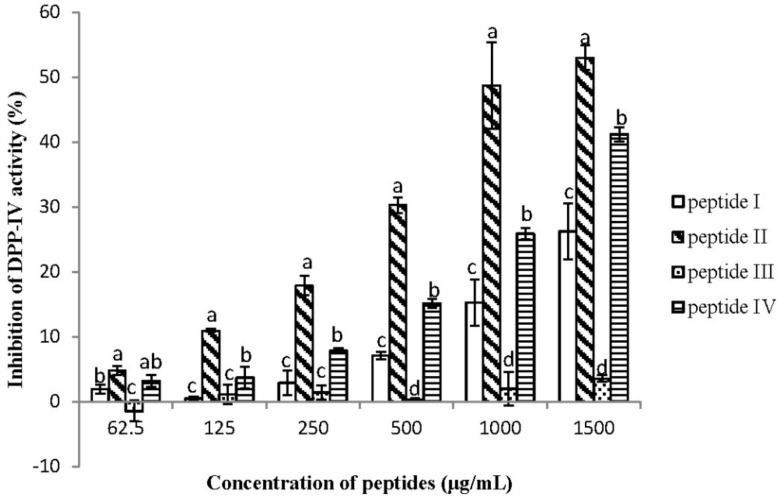
The different inhibitory capability of DPP-IV with respect to peptides I–IV. The inhibition of DPP-IV activities was determined at concentrations of 62.5, 125, 250, 500, 1000, and 1500 μg/mL respectively (*n* = 3). Values are presented as mean ± SD. Bars with different letters are significantly different at *p* < 0.05.

Four peptides were synthesized to detect the DPP-IV inhibitory activities at the concentration from 62.5 to 1500 μg/mL. The results can be seen in Figure 2—four peptides all showed dose-dependent effects. Among them, peptide II showed much higher inhibitory activity at both low and high concentrations. Almost 50% of DPP-IV enzyme activity was inhibited when treated with 1000 μg/mL of peptide II. On the other hand, Peptide III showed almost no inhibition at the concentration below 1000 μg/mL; then, its activity increased with increasing concentrations, but still showed the lowest activity at 1500 μg/mL. The inhibitory activity of peptide IV was similar to peptide II at 62.5 μg/mL, but was surpassed by peptide II with increasing concentration. In addition, the DPP-IV inhibition of peptide I was lower than peptide IV, but higher than peptide III.

### 2.3. Effects on Glycated Hemoglobin and Secretion of Glucagon and Insulin

**Figure 3:**
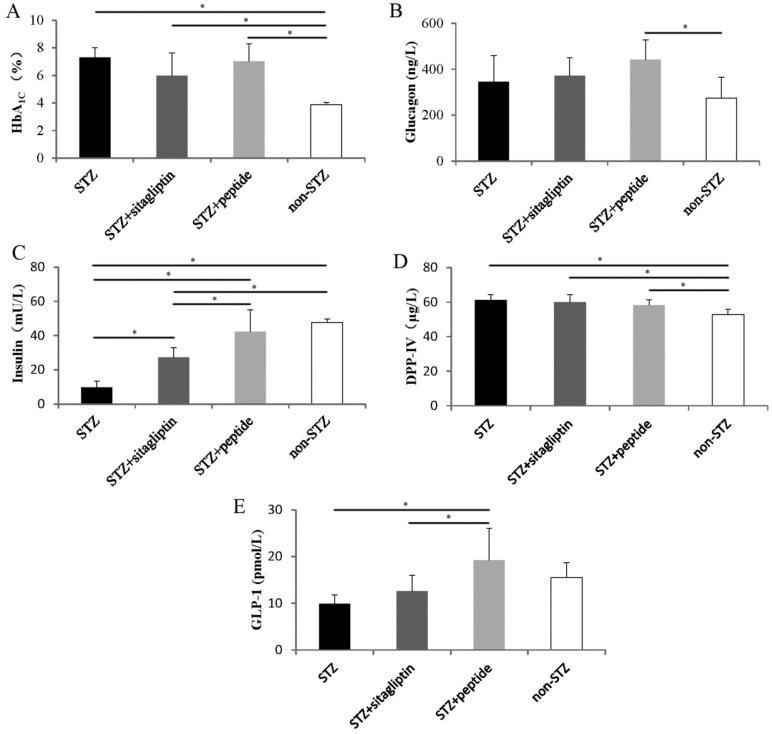
Effect of daily DPP-IV inhibitor administration on the (**A**) HbA1c levels, (**B**) plasma glucagon levels, (**C**) plasma insulin levels at day 57, (**D**) DPP-IV concentrations, and (**E**) glucagon-like peptide-1 (GLP-1) concentrations. The black columns represent the STZ control mice (*n* = 9); the deep gray columns show STZ mice administrated with sitagliptin (*n* = 6); the light gray columns represent STZ mice administrated with synthetic peptide II (*n* = 7); and the white columns show the normal control (*n* = 10). Data are presented as means ± SD. Statistical significance: * *p* < 0.05.

Considering the remarkable inhibitory activity of peptide II in vitro, our further studies were focused on this peptide. Synthesized peptide II was used to perform chronic administration experiments in STZ-induced hyperglycemic mice. The results of the HbA1c levels (Figure 3A) show that because of the toxic effects of STZ, the HbA1c levels in STZ-treated mice were significantly higher than those in non-STZ control mice. Treatment of peptide II for 57 days to STZ-induced mice did not show a significant difference in HbA1c levels compared to STZ control animals. However, the data of STZ and sitagliptin mice showed that the HbA1c level decreased from 7.30 ± 0.70% to 5.98 ± 1.63% compared with the level in the STZ group.

The effects of the administration of peptides and sitagliptin on the plasma glucagon levels in STZ control are presented in Figure 3B. There was no amelioration of the glucagon levels, and we observed that blood insulin increased significantly in the STZ plus peptide II mice (42.30 ± 12.64 mU/L), compared to the STZ-treated mice that were administered water (9.79 ± 3.62 mU/L) (Figure 3C). Moreover, the peptide improved the insulin levels more effectively than sitagliptin (27.35 ± 5.65 mU/L) at the administration dose used in the present study.

### 2.4. Effects on Blood Dipeptidyl Peptidase IV and Glucagon-Like Peptide-1 Concentrations

The concentration of DPP-IV in blood was detected using a DPP-IV ELISA kit. The DPP-IV level in STZ mice was significantly higher than that in non-STZ mice (Figure 3D). After the 57-day experiment, the concentration of GLP-1 in STZ plus peptide II mice (19.22 ± 6.79 pmol/L) was significantly higher than that in STZ control, and showed almost no significant difference from that in the normal control group (15.52 ± 3.17 pmol/L) (Figure 3E). After the gavage administration of sitagliptin, the GLP-1 concentration slightly increased, from 9.85 ± 1.96 pmol/L to 12.57 ± 3.38 pmol/L, but without any significant difference compared with the STZ control.

### 2.5. Effects on Islet Alpha-Cell and Beta-Cell

**Figure 4:**
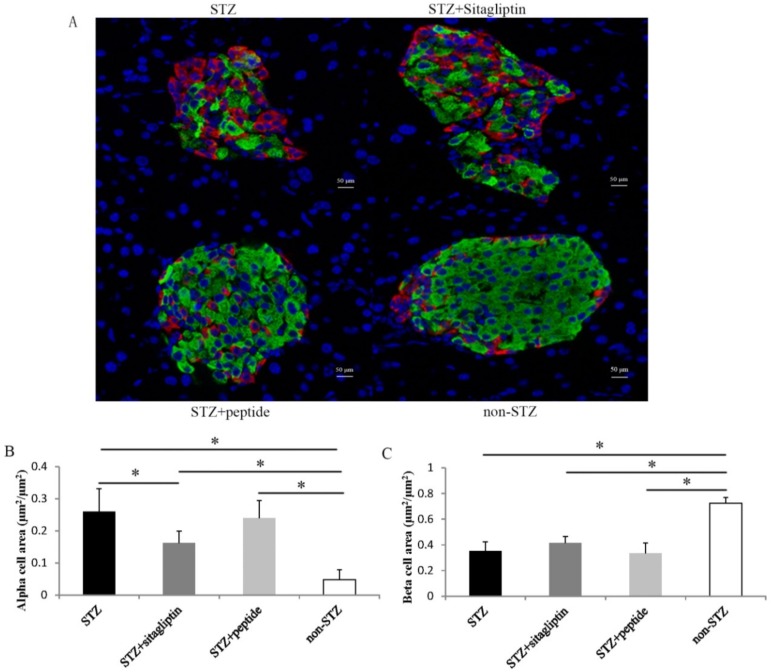
Immunochemical analysis was investigated at day 57. (**A**) Pancreatic sections were stained with insulin (green), glucagon (red), with representative islets shown (scalebars: 50 μm; magnification: ×400). (**B**) Glucagon-positive (alpha) cell area per islet area. (**C**) Insulin-positive (beta) cell area per islet area. The data of the alpha and beta cell area are presented as means ± SE (Standard Error). Black columns show STZ control mice (*n* = 9); the deep gray columns show the STZ mice administrated with sitagliptin (*n* = 6); the light gray columns represent STZ mice administrated with synthetic peptide II (*n* = 7); and the white columns show the normal control (*n* = 10). Every islet executed three slices. Statistical significance: * *p* < 0.05.

To evaluate the effect of the DPP-IV inhibitory peptide on islet morphology in STZ-treated mice, we examined insulin and glucagon double immunofluorescence staining in the pancreas (Figure 4A). In non-treated STZ mice, the beta-cell area decreased by approximately 50%, and the alpha-cell area increased 5.4-fold compared with non-STZ mice. However, treatment with peptide and sitagliptin significantly improved this condition and decreased the alpha-cell area. In sitagliptin-treated mice, the alpha-cell area significantly decreased by 37.8% compared with that in STZ-treated mice (Figure 4B). The alpha-cell area in the peptide-treated group was also lower than that in the STZ control group, but not significantly. In contrast, the effects of peptide II and sitagliptin on the beta-cell area were not obvious (Figure 4C).

## 3. Discussion

Research on DPP-IV inhibitory peptides from food protein sources has become popular, but the number of peptide sequences identified to date is limited. The sequences of amino acids are inactive within the sequence of the parent protein, but exhibit a bioactive effect once released by endogenous enzymes during food processing, microbial fermentation, or chemical and enzymatic hydrolysis [32]. These peptides vary widely in terms of their length (2–17 amino acids long), amino acid composition, and potency (half-maximal inhibitory concentration (IC50) values) [33]. The most common way to generate novel bioactive peptides is by enzymatic hydrolysis of protein substrates using food-grade enzymes. In this study, we tried to discover the bioactive peptides from black tea, because it can be hydrolyzed by endogenous enzymes and chemical reactions during the fermentation process of manufacture.

Methods including ultrafiltration, ion-exchange chromatography, size-exclusion chromatography, and reversed-phase high-performance liquid chromatography (RP-HPLC) usually used to purify bioactive peptides. However, the separation and detection of small peptides from black tea extraction presented an analytical challenge because of the presence of phenolic compounds. Because polyphenols can bind to the peptides and form soluble complexes, or even insoluble particles [34,35], we chose SDS-PAGE, which is conveniently coupled with LC-MS/MS to purify and identify the peptides. The peptides remained aggregated at ~15 kDa, as shown in Figure 1, though they have been treated with ultrafiltration, using membranes with a molecular mass cutoff of 1 kDa. This might be caused by the fact that black tea infusions will produce cloudy coacervates on cooling, which are known as tea cream [36]. To verify this hypothesis, the solution was incubated at 70 °C after dissolving lyophilized compounds of 1 kDa fractions; then, Tricine–SDS-PAGE performed (shown in Figure S3). The results showed that peptides <1 kDa were successfully observed in the gel of Lapsang souchon as well as the other two black tea samples collected from Zhejiang and Yunnan Provinces.

**Figure 5:**
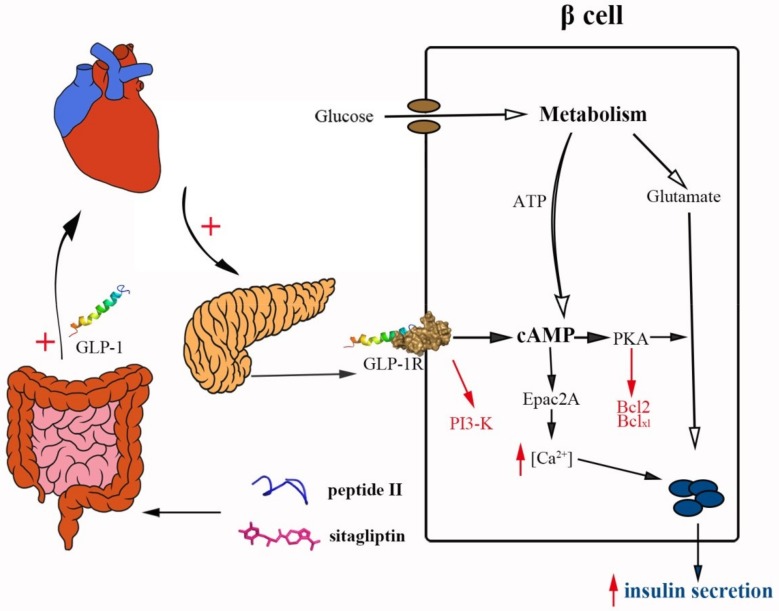
The role of peptide II and sitagliptin as dipeptidyl peptidase-IV (DPP-IV) inhibitors. Glucagon-like peptide-1 (GLP-1) is released from the enteroendocrine cells in the gastrointestinal tract, then diffuses into the capillaries where degradation by DPP-IV and circulating in the plasma begins. Further degradation occurs during passage through the liver, as well as on the way to pancreatic islet. The intake of peptide II and sitagliptin inhibits the activity of DPP-IV and remains in the concentration of GLP-1 in plasma. The GLP-1 receptor activation in the pancreatic beta-cells leads to insulin secretion via the stimulation of incretin/cAMP pathways and recruits signaling mechanisms, leading to cell survival. The figure is reproduced from [40,41,42].

While the DPP-IV inhibitory activities of food-derived peptides have been shown in numerous in vitro studies, the literatures on their effects in vivo remain sparse; however, some promising results have also been reported [37,38,39]. In our study, after STZ treatment, the blood concentrations of DPP-IV in mice increased and the concentrations of GLP-1 decreased (Figure 3D,E). After 57 days, both the peptide II- and sitagliptin-treated mice showed increased GLP-1 concentrations (9.3 and 2.7 pmol/L, respectively) and plasma insulin levels (4.3- and 2.7-fold, respectively). As shown in Figure 5, the degradation of GLP-1 in vivo was extremely rapid, and over half of the newly secreted GLP-1 degrades even before it is secreted from the gut. Further degradation occurs during the passage through the liver, and only a small fraction of the originally secreted GLP-1 fraction actually reaches systemic circulation in the intact form [40]. The effect of GLP-1 on insulin secretion occurs only at elevated glucose concentrations, and is mediated through cyclic adenosine monophosphate (cAMP) signaling. The engagement of GLP-1 and the GLP-1 receptor (GLP-1R) stimulates cAMP formation, and the downstream pathways involve both protein kinase A (PKA) and cAMP–guanine nucleotide exchange factor II (Epac2)-dependent pathways. Glutamate, derived from the malate–aspartate shuttle upon glucose stimulation, amplifies insulin granule exocytosis by cAMP/PKA signaling [41]. Moreover, GLP-1 produces a rapid increase in intracellular calcium, which also supports the exocytotic response [42]. Therefore, the increase of insulin can be explained by the enhancement of GLP-1 levels.

There is no doubt that food-derived bioactive peptides influence insulin stimulation. Whether these peptides have a beneficial effect on islets, similar to other DPP-IV inhibitors [11,43], is of great interest. In diabetes, both inappropriate glucagon secretion, which is attributed to alpha-cells, and impaired insulin secretion, which is attributed to beta-cells, contributed to hyperglycemia. STZ is a type of beta-cell toxin, and the area of beta-cells was significantly reduced after the injecting STZ (Figure 4A,B). Studies have shown that GLP-1 has antiapoptotic action mediated by cAMP- and phosphatidylinositide 3-kinase (PI3K)-dependent signaling pathways [44]. The stimulation of cAMP by GLP-1 increased the expression of antiapoptotic proteins Bcl-2 and Bcl-xL, and the activation of PI3K was required to prevent proapoptotic events in cells exposed to H_2_O_2_. However, only slight proliferation was observed after peptide II administration in the present study (Figure 4A,C). The concentration of insulin in the blood dramatically increased, indicating the improvement of the beta-cell capacity. Thus, DPP-IV inhibition with peptide II may protect beta-cell mass via an antiapoptotic mechanism, rather than via the induction of cell proliferation. However, GLP-1 has no direct effect on alpha-cells, as GLP-1 receptors on pancreatic alpha-cells are scarce or nonexistent [45]. In the present study, alpha-cell proliferation was accompanied by beta-cell loss, and suppressed by the administration of sitagliptin and peptide II. Takeda et al. proposed that the local insulin deficit in islets leads to pancreatic alpha-cell proliferation [13]. Therefore, the enhancement of insulin levels might contribute to the reduction of alpha-cell proliferation.

Despite the positive effect of peptide II on stimulating insulin secretion and reducing alpha-cell proliferation, no significant difference was found in HbA_1c_ levels among the three STZ-treated groups. This might be because of the method used to make hyperglycemic mice in our study: treating them with STZ (50 mg/kg, BW) for three successive days. According to study of Elizabeth R. Gilbert et al., C57BL/6 mice treated with a low-fat diet and an injection of STZ (40 mg/kg, Body Weight) for three days showed significant insulin tolerance compared to low-fat diet treated mice without an STZ injection [46]. The STZ-treated mice in our study might also suffer serious insulin resistance, and the increasing insulin levels in the peptide II-treated group are not enough to improve the HbA_1c_ levels.

**Figure 6:**
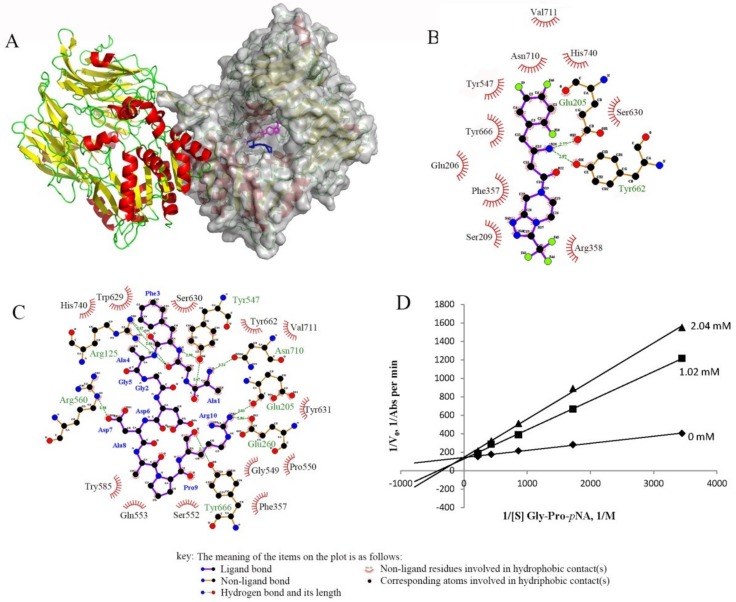
(**A**) Molecular modeling of the interaction between DPP-IV (1X70) and its ligands: sitagliptin (magenta) and peptide II (blue). Chain B is shown as a surface in gray. The profiles of the binding sites of DPP-IV with (**B**) sitagliptin and (**C**) peptide II produced by LigPlot+ show the detailed interaction. (**D**) Lineweaver–Burk plot for DPP-IV activity in the absence and presence of peptide II with different inhibitory concentrations (0, 1.02, and 2.04 mM).

Both in vitro and in vivo studies have shown the inhibitory effect of peptide II towards DPP-IV. The DPP-IV enzyme consists of a unique eight-bladed beta-propeller domain in the N-terminal region, and a serine protease domain in the C-terminal region [47]. Peptide II and sitagliptin are both located inside the DPP-IV large cavity, as shown in Figure 6A. Sitagliptin interacts with Glu205 and Tyr662 by hydrogen bonds in chain B of DPP-IV and some hydrophobic contacts (Figure 6B). Peptide II is also located in chain B and shows hydrogen bonding with Glu205, but the other contacts are different. The S1 pocket of DPP-IV contains Tyr631, Val656, Trp659, Tyr662, Tyr666, and Val711, and the S2 pocket contains Arg125, Glu205, Glu206, Phe357, Ser209, and Arg358 [48]. Hydrogen bonds between peptide II and the S1 (Tyr666) and S2 (Arg125, Glu205) of the DPP-IV enzyme can be seen in Figure 6C. Thus, peptide II might act as a competitor like sitagliptin by blocking the binding of other substrates, such as GIP and GLP-1. An enzyme kinetic experiment was then performed to check the inhibition patter of peptide II, and the Lineweaver–Burk plot in Figure 6D shows that it was a competitive inhibitory mode, which was in accordance with the docking result.

## 4. Materials and Methods

### 4.1. Chemicals and Materials

The enzyme, human recombinant DPP-IV (Lot: D4943-1VL), expressed in baculovirus-infected S/9 cells, the substrate, Gly-Pro *p*-nitroanilide (Gly-Pro *p*NA; Lot: G0513), and streptozotocin (STZ; Lot: V900890) were purchased from Sigma-Aldrich (St. Louis, MO, USA). The antidiabetic drug sitagliptin (Januvia 100 mg; Merk Sharp and Dohme Ltd., Hertfordshire EN11 9BU, Hertford Road, Hoddesdon, UK) was purchased at a drugstore in China. Other reagents, such as Folin–Ciocalteu (Lot: 73104861), gallic acid (Lot: XW01499171), and Coomassie Blue R-250 (Lot: 71011381) were purchased from Sinopharm Chemical Reagent Co., Ltd. (Shanghai, China). Lapsang souchon (LS), a typical Chinese black tea, was obtained from a tea market in Hangzhou.

### 4.2. Extraction

A total of 2 g (±0.01) of dry tea leaves were ground into powder and extracted with 150 ml of boiling ultrapure water (Millipore, Kenilworth, NJ, USA in a water bath at 100 °C for 45 min. After suction filtration, the extracts were diluted with ultrapure water to 200 mL. Lapsang souchon peptides were crudely separated by ultrafiltration using membranes with a molecular mass cutoff of 50, 10, 5 kDa, and 1 kDa, and 100 mL fractions were collected, lyophilized, and stored in a desiccator at –20 °C.

The lyophilized compounds were subsequently dissolved in 2 mL of ultrapure water (Millipore) respectively when they were used. The total tea phenol (TP) content was determined using 10% Folin–Ciocalteu reagent, as previously described [49]. The 1 kDa membrane filtrate was resolved by SDS-PAGE, using 12% acrylamide gels stained with colloidal Coomassie Blue R-250 [50]. The peptides were extracted from the gel for further analysis.

### 4.3. Identification of the Peptide Sequence by LC-MS/MS Analysis

The identification of amino acid sequences was assisted by PTM Biolabs, Inc. (Hangzhou, China). The peptides were dissolved in 0.1% formic acid (FA) and loaded onto a reversed-phase pre-column (Acclaim PepMap 100, Thermo Scientific, Waltham, MA, USA). Peptide separation was performed using a reversed-phase analytical column (Acclaim PepMap RSLC, Thermo Scientific) with a linear gradient of 6–22% solvent B (90% ACN, 10% H_2_O, 0.1% FA) for 22 min, 22–36% solvent B for 10 min, and 36–80% solvent B for 5 min, at a constant flow rate of 300 nl/min on an EASY-nLC 1000 UPLC (Ultra Performance Liquid Chromatography) system. Solvent A was comprised of 0.1% FA and 2% ACN (Acetonitrile). The resulting peptides were analyzed by the Q Exactive Hybrid Quadrupole-Orbitrap Mass Spectrometer (Thermo Scientific). Intact peptides were detected in the Orbitrap at a resolution of 70,000. Peptides were selected for MS/MS using 28% normalized collisional energy (NCE); ion fragments were detected in the Orbitrap at a resolution of 17,500. A data-dependent procedure that alternated between one MS scan, followed by 20 MS/MS scans, was applied for the top 20 precursor ions above a threshold ion count of 2E4 in the MS survey scan, with 30.0 s dynamic exclusion. The electrospray voltage applied was 2.0 kV. Automatic gain control (AGC) was used to prevent overfilling of the ion trap; 5E4 ions were accumulated for generation of MS/MS spectra. For MS scans, the *m*/*z* scan range was 350 to 1800.

### 4.4. Dipeptidyl Peptidase IV Inhibitory Assay and Kinetics Assays

The DPP-IV inhibitory activities of the substance after ultrafiltration and the peptides identified (synthesized by Chinese Peptide Company, Hangzhou, China; purity of 96–97%) were evaluated. The DPP-IV inhibition assay was performed as described by Velarde-Salcedo et al. [50], with slight modifications. The assay was performed in 96-well microplates, and 100 μL of enzyme (final concentration 1.9 U/L) was preincubated with the samples (the concentrated ultra-filtrate mentioned above and the synthesized peptide, with concentrations from 62.5–1500 μg/mL) at 37 °C for 15 min prior to the initiation of the reaction, by adding 50 μL of Gly-Pro p-nitroanilide (final concentration 0.29 mM). The final volume of the reaction system was 200 μL, and the reaction was conducted in 100 mM Tris-HCl buffer (pH 7.6). The absorbance was obtained at 405 nm in a microplate reader (Synergy H1, BioTek Instruments Inc., Winooski, VT, USA), after incubation at 37 °C for 1 h.

The most potent peptide (peptide II) was synthesized and further studied for its modes of action on DPP-IV using kinetics assays. The initial rate of hydrolysis of the substrate Gly-Pro p-nitroanilide by DPP-IV was measured using substrate concentrations of 0.29, 0.58, 1.16, 2.32, and 4.64 mM (final assay concentrations) in the absence (100 mM Tris-HCl buffer, pH 7.6) and presence of different concentrations of the peptide II (1.02 and 2.04 mM, respectively). The substrate (50 μL), with or without an inhibitor (50 μL), was pre-incubated at 37 °C for 15 min, and the reaction was started by the addition of 100 μL of the DPP-IV enzyme (final concentration: 1.9 U/L). The absorbance of the released p-nitroanilide was monitored at 37 °C and 405 nm for 60 min using a microplate reader (Synergy H1, BioTek Instruments Inc.). Lineweaver–Burk plots of DPP-IV activity in the absence and presence of peptide II were created to show the modes of inhibition.

### 4.5. Docking Analysis of Dipeptidyl Peptidase IV with Peptide II

Rigid body docking analysis of the peptide II and DPP-IV (Protein Data Bank code: 1X70) enzymes were performed using ClusPro 2.0 (http://cluspro.bu.edu) [51,52,53,54]. Models of peptides II was built using the I-TASSER server, which employs a fragment-based method. Peptide fragments were obtained from multiple template structures, and reassembled based on threading alignments. The best ranking model for peptide II, as judged from the C-score value, was selected for docking simulations [55,56,57]. LigPlot^+^ (v.1.4.5, European Bioinformatics Institute) was used to observe the interactions in detail [58]. The interaction between sitagliptin and DPP-IV was obtained from the information from PDB code 1X70.

### 4.6. Animals

Male ICR mice at the age of four weeks were obtained from the Laboratory Animal Center of Zhejiang University (Hangzhou, China) and housed under a 12 h light/dark cycle with free access to food and water. The animal protocol was approved by the Committee on the Ethics of Animal Experiments of Zhejiang University (Permit Number: SYXK 2018-0001, 3 January 2018), and all experiments were performed in accordance with Zhejiang University Laboratory Animal Center Guidelines and the Regulations for Laboratory Animals Care and Usage.

### 4.7. Research Design and Treatment

The mice were divided into four groups: non-STZ, STZ, STZ plus sitagliptin, and STZ plus peptide II. The mice were injected with STZ (50 mg/kg) dissolved into sodium citrate buffer (pH 4.5) for three successive days to induce hyperglycemia. Every group contained 10 mice. Subsequently, the mice respectively received gavage with ultrapure water, sitagliptin (4 mg/kg), and synthesized peptide II (400 mg/kg), according to the grouping every day from the fourth day after injection (Day 1). The doses of sitagliptin and peptide II were set according to research of Huang et al. [39], Hsieh et al. [37], and Uenishi et al. [38], as well as our preliminary experiment.

### 4.8. Biochemical Determinations

At Day 57, the animals were sacrificed, and blood samples were collected and mixed with 10% cooled 20 mg/ml EDTA-2K (Ethylene Diamine Tetraacetic Acid Dipotassium) to detect glycated hemoglobin (HbA_1c_). Blood samples for the other biochemical determinations were also collected in tubes, centrifuged (3000× *g*, 15 min), and subsequently stored at −80 °C. The levels of HbA_1c_ were measured using an automatic analyzer MQ-2000pt produced by Medconn Medical Technology Co. (Shanghai, China). Plasma insulin, glucagon, GLP-1, and DPP-IV concentrations were measured using a mouse ELISA kit (Shanghai Lengton Bioscience Co., Ltd., Shanghai, China).

### 4.9. Immunohistochemistry and Islet Morphometry

The pancreases were resected immediately after the mice were sacrificed and fixed in 4% (*w*/*v*) formaldehyde. The immunohistochemistry procedures were performed according to Y. Takeda et al. [13]. The stained sections were observed by an inverted fluorescence microscopy (NIKON ECLIPSE TI-SR; Nikon Corporation, Tokyo, Japan), and digital images were collected using Nikon imaging system (NIKON DS-U3). The insulin- and glucagon-positive cell areas were respectively measured by using Image-Pro Plus 6.0 (Media Cybernetics, Inc., Rockville, MD, USA).

### 4.10. Statistical Analysis

The statistical analysis of the data was performed using SAS (Statistical Analysis System), University Edition (Institute Inc., Cary, NC, USA). In vitro experiments were designed with three replications. A general linear model (GLM) and Ryan–Einot–Gabriel–Welsch multiple range (REGWQ)’s tests (*p* < 0.05) were performed to detect significant differences. The data of pancreatic cell areas are presented as the means ± SE, and other data are presented as the means ± SD.

## Figures and Tables

**Table 1:** The inhibitory ratio on dipeptidyl peptidase IV (DPP-IV) by ultrafiltrate of Lapsang souchon from different membranes (*n* = 3). General linear model (GLM) followed by Ryan–Einot–Gabriel–Welsch’s (REGWQ’s) multiple range test. Means with different letters (a, b, c) have significant difference (*p* < 0.05).

Membrane (kDa)	Total Phenols (μg/mL)	Inhibition of DPP-IV Activity (%)
50	49.2 ± 0.83 a	75.5 ± 0.47 a
10	42.8 ± 0.59 b	73.8 ± 0.54 a
5	42.5 ± 0.42 b	75.7 ± 1.00 a
1	30.7 ± 0.82 c	72.7 ± 2.57 a

**Table 2:** Peptides <1 kDa obtained from Lapsang souchon.

No.	Peptide Sequence	pI/MW ^a^
I	QTDEYGNPPR	4.37/1176.21
II	AGFAGDDAPR	4.21/976.01
III	IDESLR	4.37/731.80
IV	IQDKEGIPPDQQR	4.56/1523.67

^a^ Theoretical data; MW (Molecular Weight) in Da.

## Supplementary Materials

Supplementary materials can be found at http://www.mdpi.com/1422-0067/20/2/322/s1.
