# Supplementary material for: A Novel Dipeptidyl Peptidase IV Inhibitory Tea Peptide Improves Pancreatic β-Cell Function and Reduces α-Cell Proliferation in Streptozotocin-Induced Diabetic Mice
Source: Int J Mol Sci. 2019 Jan 14;20(2):322. doi: 10.3390/ijms20020322 (PMC6359713; doi:10.3390/ijms20020322)
Supplement: Supplementary file 1 [file ijms-20-00322-s001.pdf]

Supplementary materials

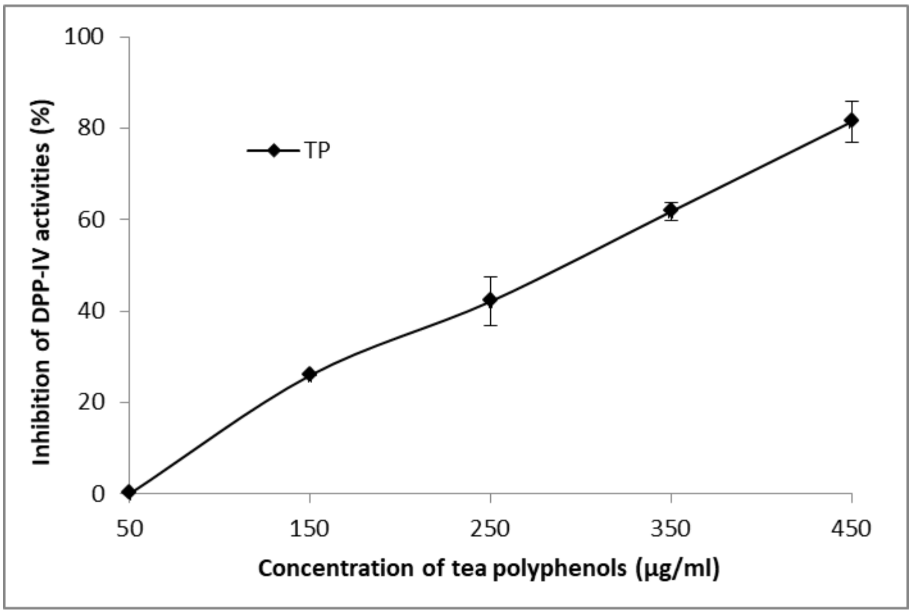

Figure S1: The inhibitory capability of DPP-IV with respect to TP was shown, the IC_{50} calculated by Microcal(TM) Origin Pro Version 8.0 (Microcal Software,Inc. Northampton, MA 01060 USA) of TP was 284.35±8.98 μg/ml. (TP = tea polyphenol; DPP-IV = dipeptidyl peptidase- IV)

a) Sequence: QTDEYGNPPR, Charge: +2, Monoisotopic m/z: 588.77026 Da (+2.61 mmu/+4.44 ppm), MH+: 1176.53325 Da, RT: 5.72 min,

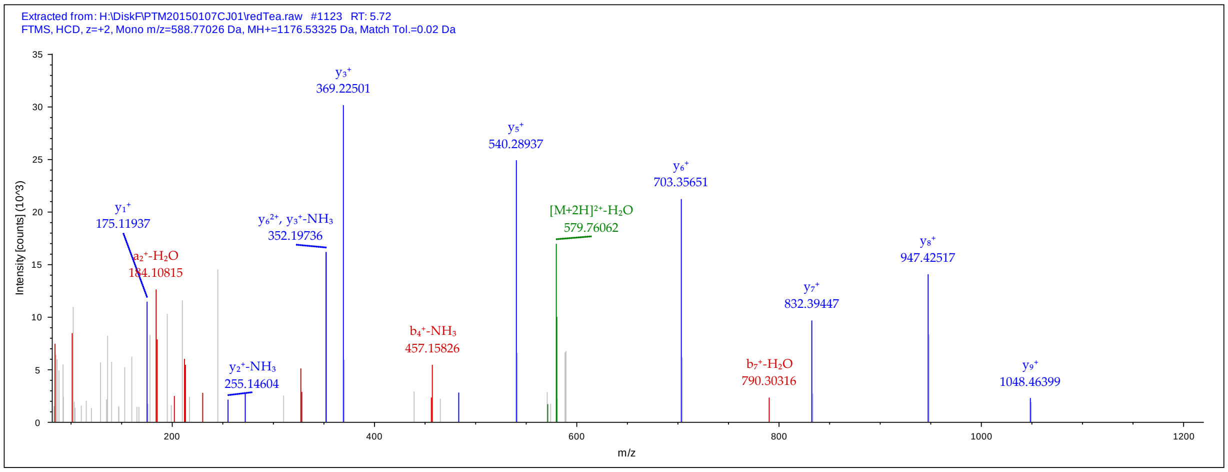

b) Sequence: AGFAGDDAPR, Charge: +2, Monoisotopic m/z: 488.73093 Da (+3.12 mmu/+6.38 ppm), MH+: 976.45458 Da, RT: 7.60 min,

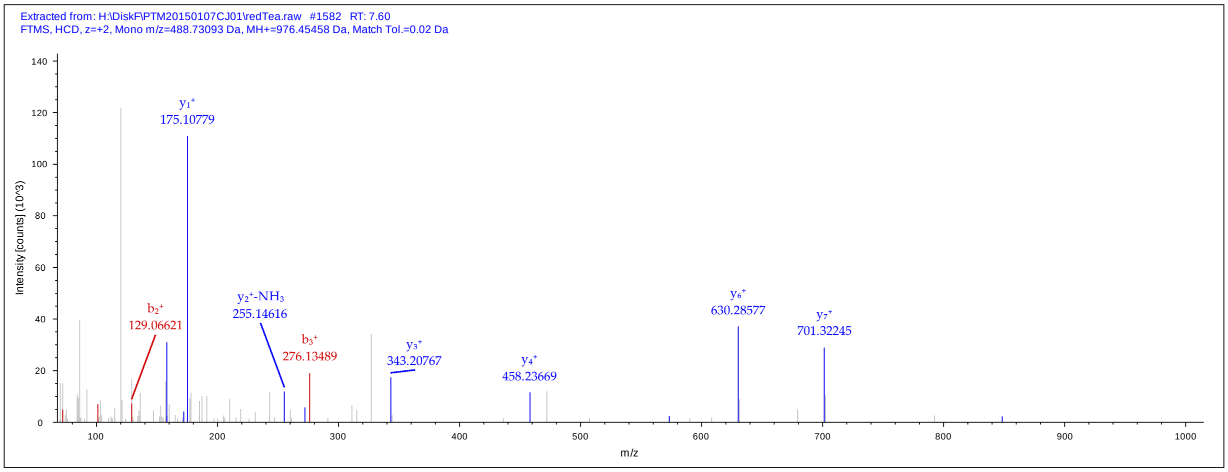

c) Sequence: IDESLR, Charge: +2, Monoisotopic m/z: 366.70026 Da (+2.28 mmu/+6.22 ppm), MH+: 732.39324 Da, RT: 5.34 min,

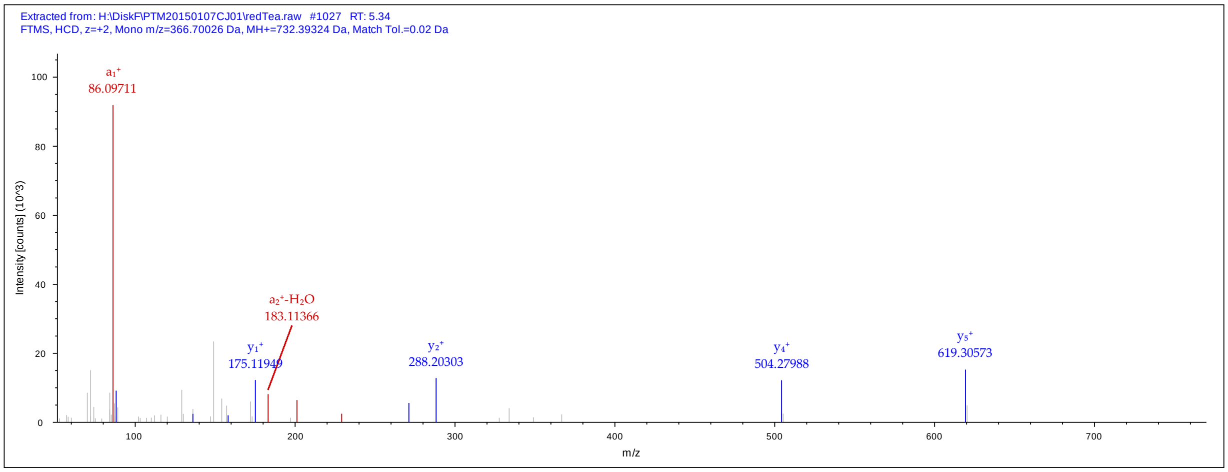

d) Sequence: IQDKEGIPPDQQR, Charge: +3, Monoisotopic m/z: 508.60190 Da (+3.28 mmu/+6.44 ppm), MH+: 1523.79114 Da, RT: 7.64 min,

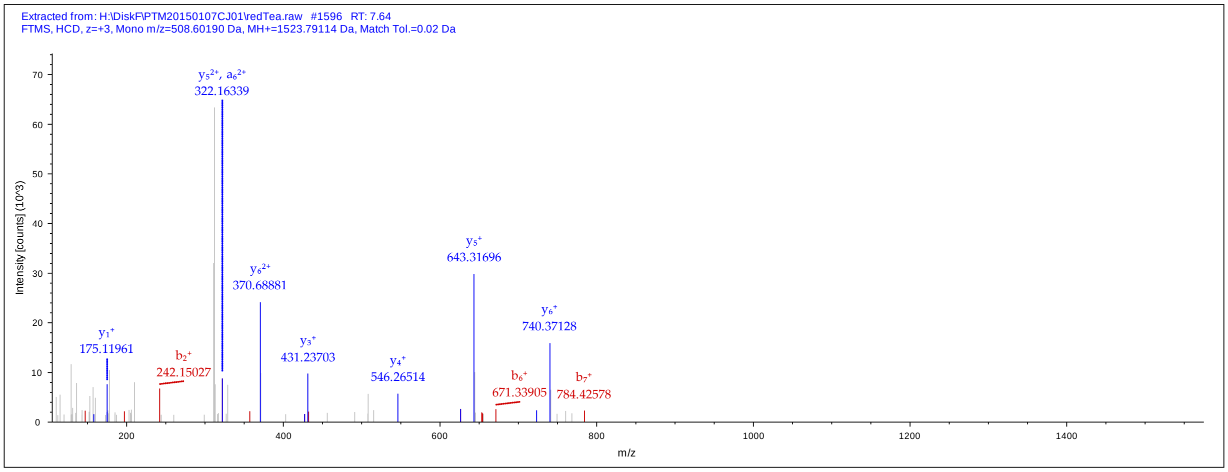

Figure S2: MS/MS chromatograms of a) peptide I; b) peptide II; c) peptide III and d) peptide IV.

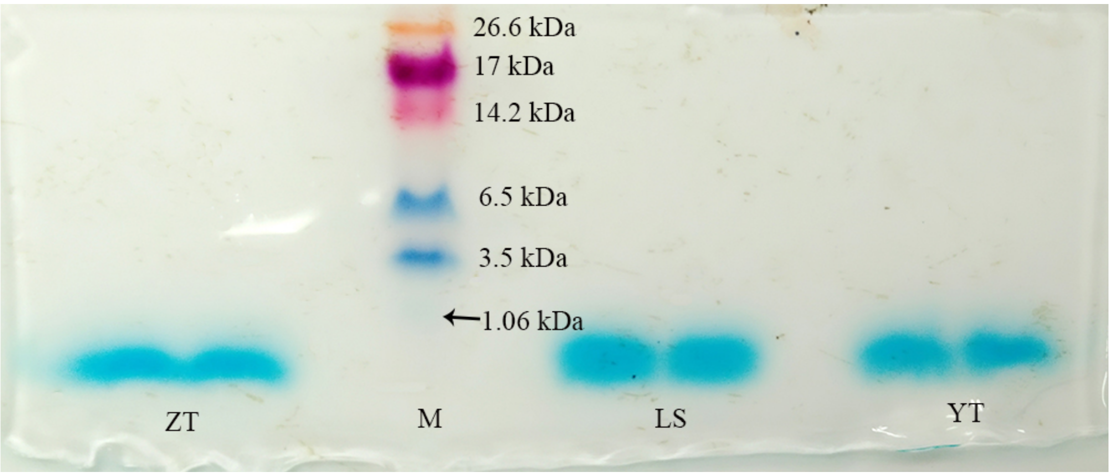

Figure S3: Tricine-SDS-PAGE was performed as described by Schaegger, H. [1].M=Marker, LS= Lapsang souchon, ZT= Black tea from Zhejiang Province of China, YT= Black tea from Yunnan Province of China.

1. Schaegger, H. Tricine-SDS-PAGE. Nat Protoc 2006, 1, 16-22, doi:10.1038/nprot.2006.4.
